# Supplementary material for: Small Molecule Modulation of MHC‑I Surface Expression: A Click Chemistry-Based Discovery Approach
Source: J Med Chem. 2025 Oct 27;68(21):22301–11. doi: 10.1021/acs.jmedchem.5c01149 (PMC12621194; doi:10.1021/acs.jmedchem.5c01149)
Supplement: Supplementary file 1 [file jm5c01149_si_001.pdf]

Supporting Information

Small Molecule Modulation of MHC-I Surface Expression: A Click Chemistry-Based Discovery Approach

Sarah E. Newkirk[1,2#], Joey J. Kelly[1,2#], Mahendra D. Chordia[1,2], Yue Dou[3], Tian Zhang[3], and Marcos M. Pires[1,2*]

[1] *Department of Chemistry, University of Virginia, Charlottesville, VA 22904, United States*
[2] *Department of Microbiology, Immunology, and Cancer, University of Virginia, Charlottesville, VA 22904, United States*
[3] *Department of Biochemistry and Molecular Genetics, School of Medicine, University of Virginia, Charlottesville, VA 22904, United States*

* corresponding author: mpires@virginia.edu

[#] *S.E.N and J.J.K contributed equally to this work*

Table of Contents

**SUPPLEMENTARY FIGURES**	**S3-S15**
Figure S1. *Chemical structures for 25 compound library*	S3
Figure S2. *Flow cytometry analysis of CT26 cells treated with 25-member library at 5 µM*	S4
Figure S3. *Analytical HPLC of reaction between 9-propargyl-2-amino-6-chloropurine and 2-azido-1-(4-methoxy-phenyl)-ethanone*	S5
Figure S4. *Analytical HPLC of reaction between 9-propargyl-2-amino-6-chloropurine and 4-azido-1-butanamine*	S6
Figure S5. *Analytical HPLC of reaction between 9-propargyl-2-amino-6-chloropurine and Boc-4-azido-L-phenylalanine*	S7
Figure S6. *Chemical structures for 380 compound library*	S8
Figure S7. *Chemical structures of triazole products from azides 1-8*	S9
Figure S8. *Flow cytometry analysis of CT26 cells treated with 1 µM 9-propargyl-2-amino-6-chloropurine or a 1:10,000 dilution of CuAAC click reagents*	S12
Figure S9. *Flow cytometry analysis of CT26 cells treated with triazole products of 3, 27, 325, and 335*	S13
Figure S10. *Flow cytometry analysis of CT26 cells treated with **CliMB-325** regioisomer*	S14
Figure S11. *Length distribution of MHC-I peptides isolated by MAE from CT26 cells*	S15
**MATERIALS AND METHODS**	**S16-S18**
**Materials**	S16
**Experimental Methods**	S16-18
Mammalian Cell Culture	S16
Flow Cytometry-Based Assays	S16
MTT Cell Viability Assay	S16
B3Z T Cell Activation	S17
Molecular Docking Studies	S17
Mild Acid Elution (MAE) of MHC-I-Bound Peptides	S17
Liquid Chromatography	S17
Mass Spectrometry Data Acquisition	S18
DDA Data Analysis	S18
**SYNTHESIS AND CHARACTERIZATION**	**S19-27**
Scheme S1: *Synthesis of 9-propargyl-2-amino-6-chloropurine*	S19
Scheme S2: *High-Throughput Synthesis of Triazole-Containing BIIB021 Derivatives*	S22
Scheme S3: *Synthesis of **CliMB-325***	S23
**REFERENCES**	**S28-29**

SUPPLEMENTARY FIGURES

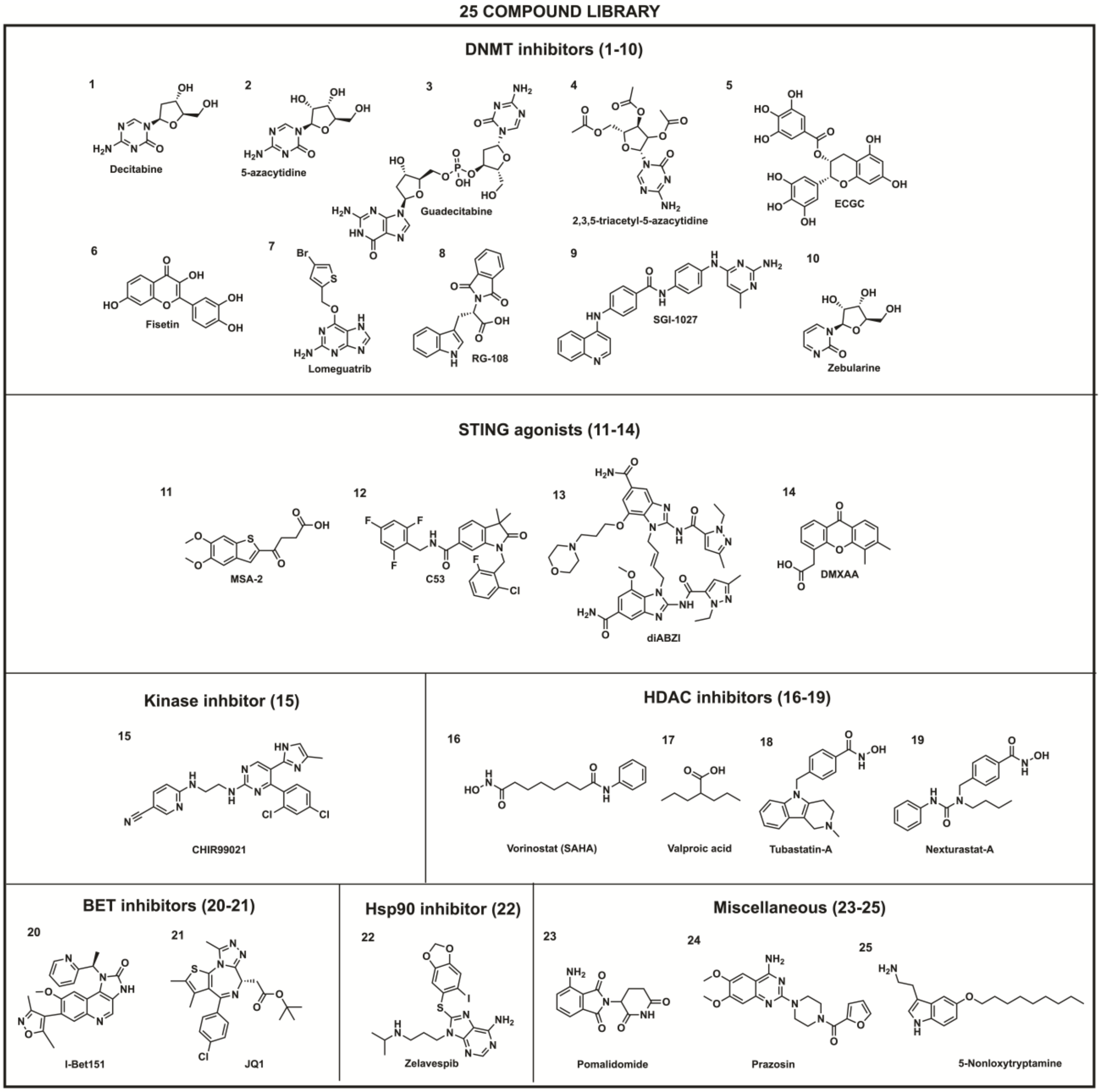

Figure S1. Structures of 25 compounds in library of small molecule MHC-I inducers, subdivided by class. Compounds 1-10 are DNMT inhibitors, 11-14 are STING agonists, 15 is a kinase inhibitor, 16-19 are HDAC inhibitors, 20-21 are BET inhibitors, 22 is an Hsp90 inhibitor, and 23-25 are miscellaneous compounds.

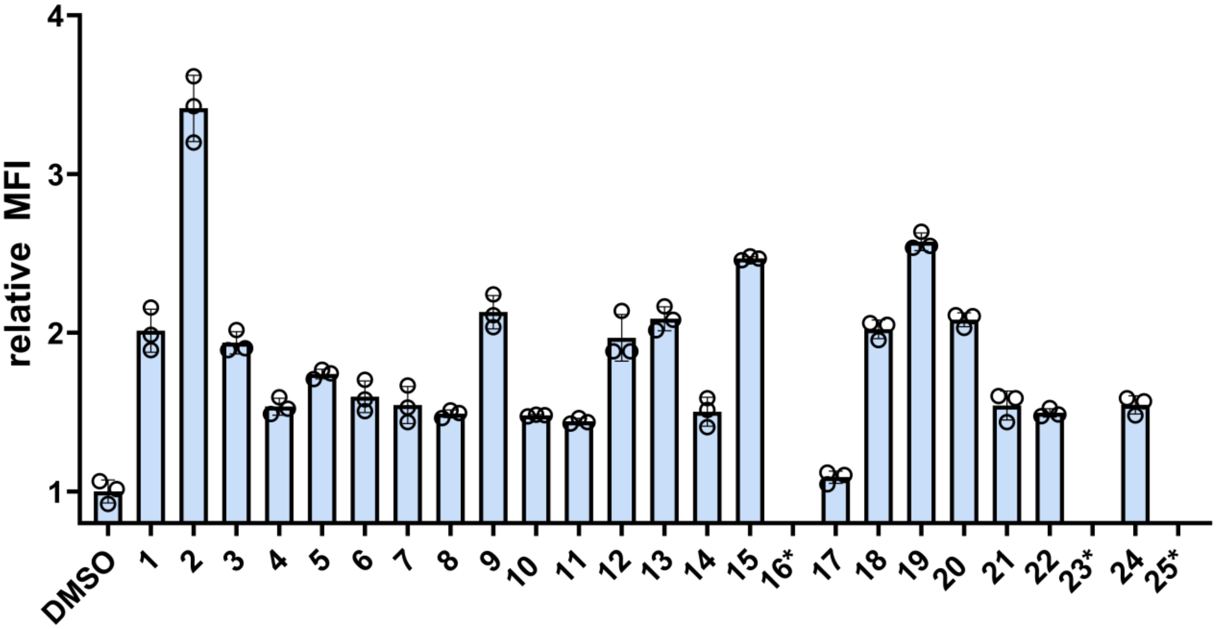

Figure S2. Flow cytometry analysis of CT26 cells treated with 25-member library at 5 μM. H-2K^d surface expression was measured by APC-conjugated anti-mouse H-2K^d antibody. MFI means fluorescence intensity of the level of fluorescence relative to the DMSO control. Compounds containing no data bar and denoted with * indicate that the compound was toxic to the cells at 5 μM concentration. Data are represented as mean ± SD (n=3).

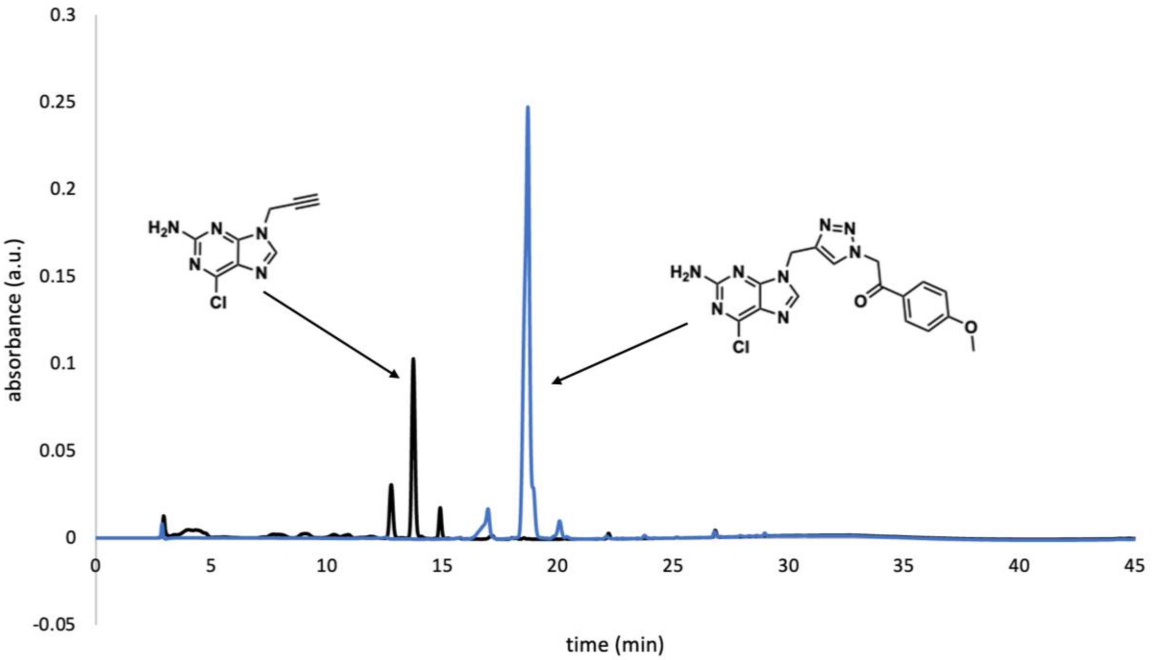

Figure S3. Analytical HPLC of reaction between 9-propargyl-2-amino-6-chloropurine and 2-azido-1-(4-methoxy-phenyl)-ethanone. 9-propargyl-2-amino-6-chloropurine and 2-azido-1-(4-methoxy-phenyl)-ethanone, each at a concentration of 10 mM, were reacted with 40 mM L-ascorbic acid and 2 mM $CuSO_4$/THPTA in a 3:2 ratio of DMSO to water, with a total reaction volume of 100 µL. Overlaid HPLC chromatograms of reaction mixture prior to addition of 2-azido-1-(4-methoxy-phenyl)-ethanone (black) and the full reaction mixture after incubation shaking at 37 ^oC for 20 h (blue) are shown.

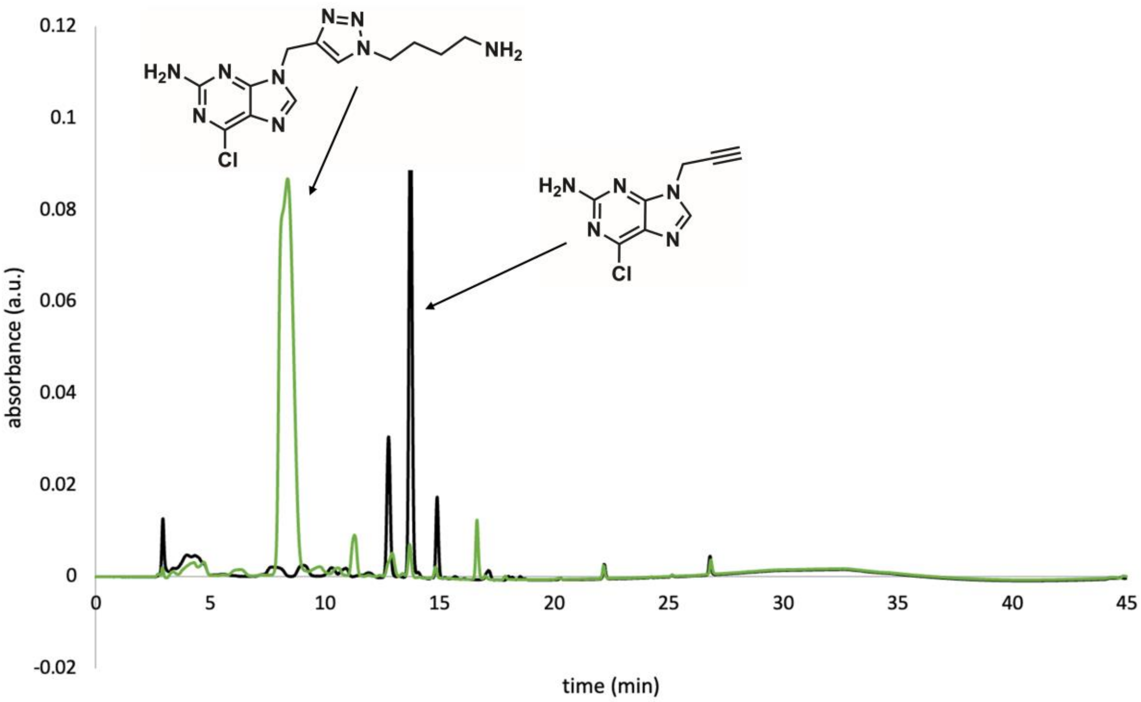

Figure S4. Analytical HPLC of reaction between 9-propargyl-2-amino-6-chloropurine and 4-azido-1-butanamine. 9-propargyl-2-amino-6-chloropurine and 4-azido-1-butanamine, each at a concentration of 10 mM, were reacted with 40 mM L-ascorbic acid and 2 mM $CuSO_4$/THPTA in a 3:2 ratio of DMSO to water, with a total reaction volume of 100 µL. Overlaid HPLC chromatograms of reaction mixture prior to addition of 4-azido-1-butanamine (black) and the full reaction mixture after incubation shaking at 37 ^{o}C for 20 h (green) are shown.

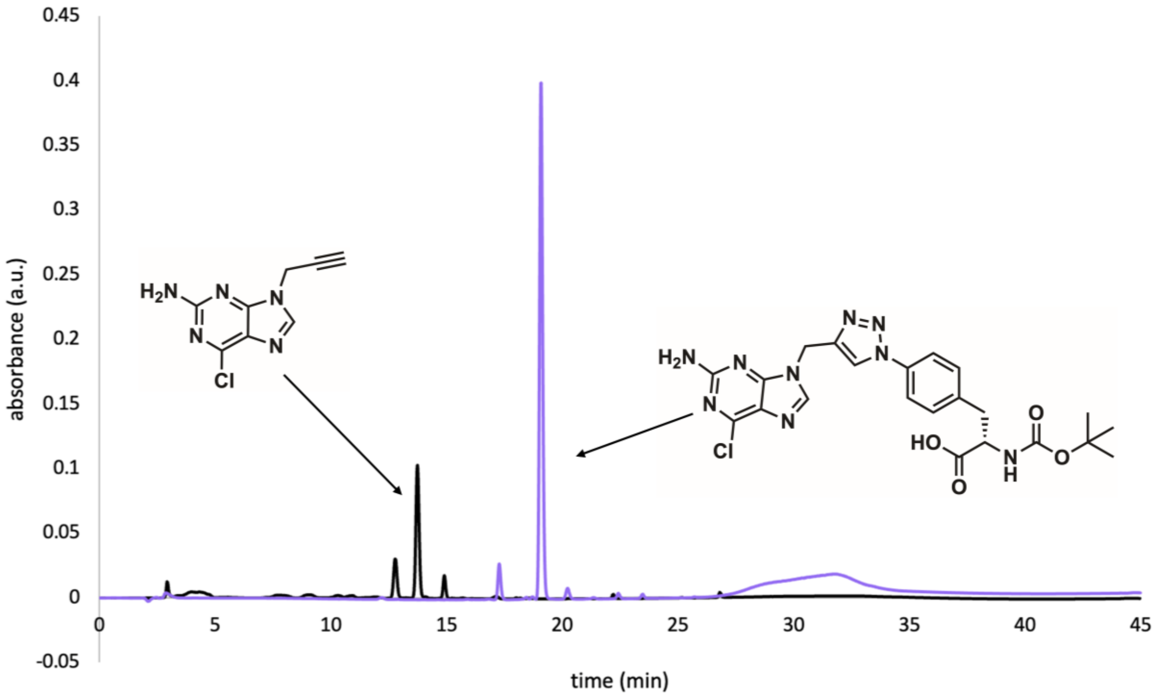

Figure S5. Analytical HPLC of reaction between 9-propargyl-2-amino-6-chloropurine and Boc-4-azido-L-phenylalanine. 9-propargyl-2-amino-6-chloropurine and Boc-4-azido-L-phenylalanine, each at a concentration of 10 mM, were reacted with 40 mM L-ascorbic acid and 2 mM $CuSO_4$/THPTA in a 3:2 ratio of DMSO to water, with a total reaction volume of 100 µL. Overlaid HPLC chromatograms of reaction mixture prior to addition of Boc-4-azido-L-phenylalanine (black) and the full reaction mixture after incubation shaking at 37 ^{o}C for 20 h (purple) are shown.

1 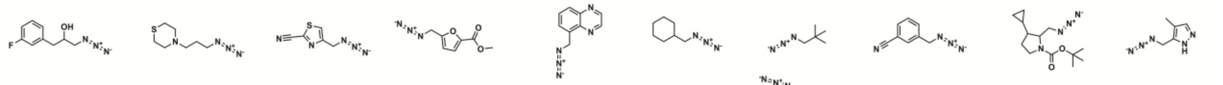

11 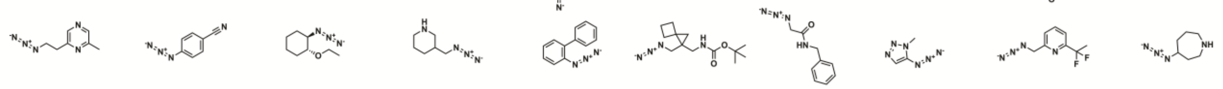

21 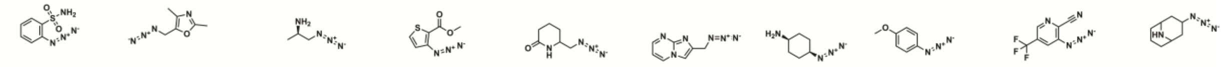

31 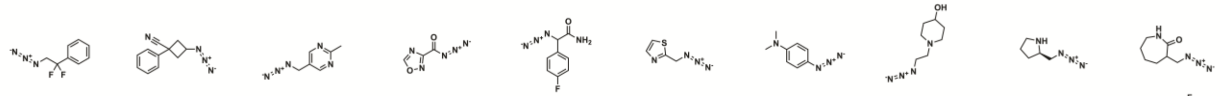

41 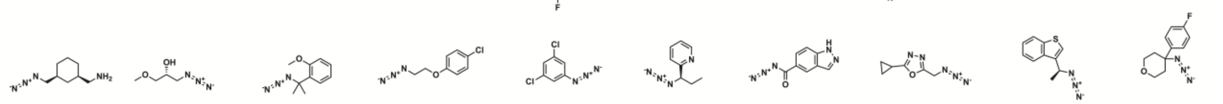

51 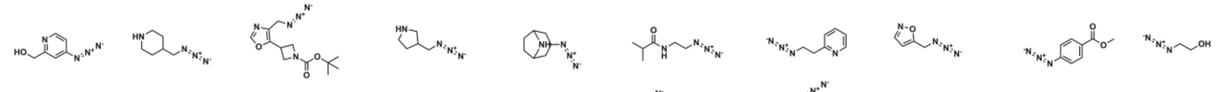

61 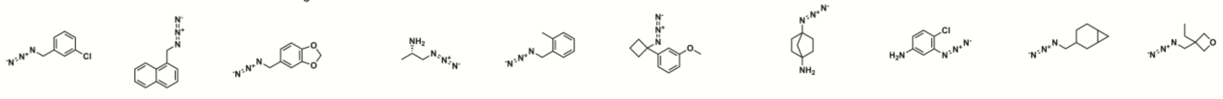

71 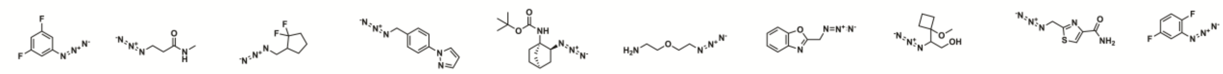

81 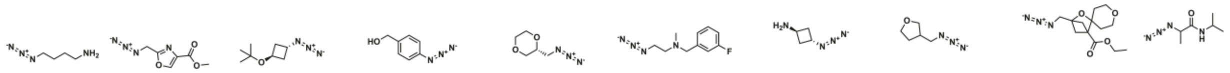

91 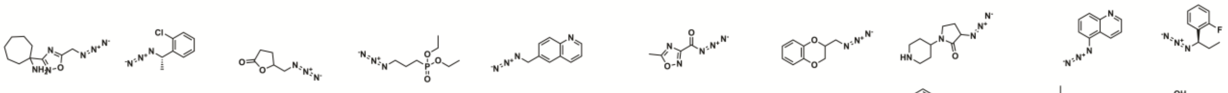

101 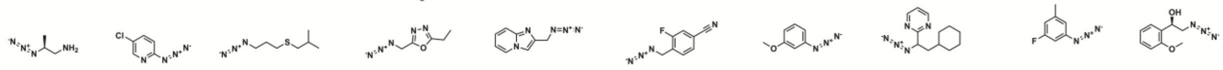

111 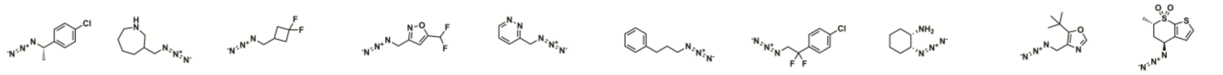

121 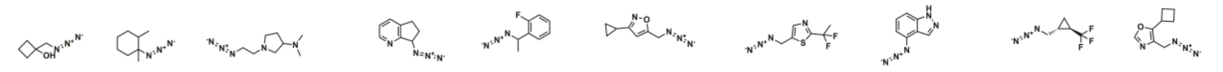

131 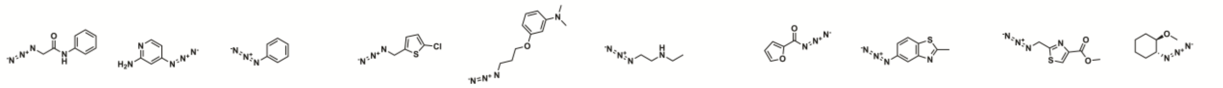

141 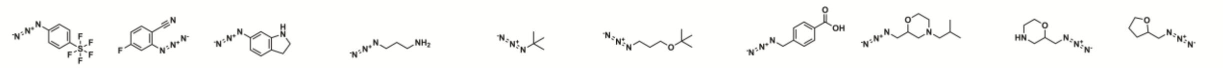

151 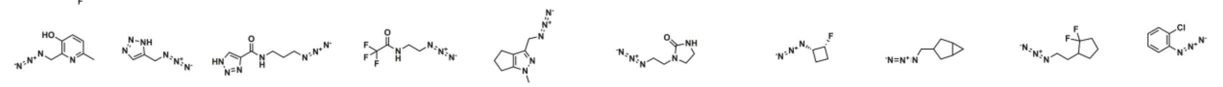

161 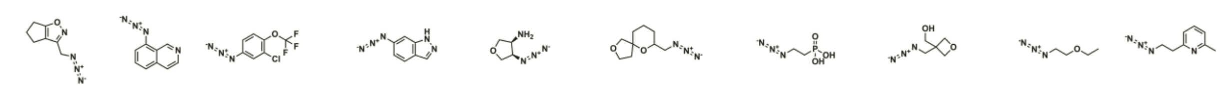

171 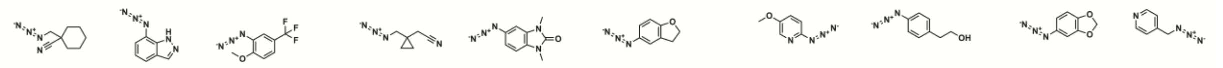

181 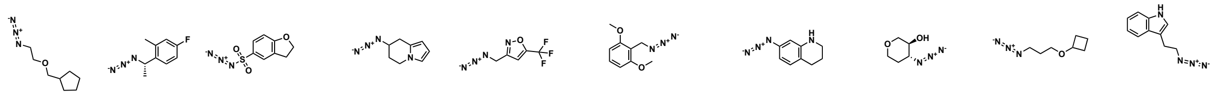

191 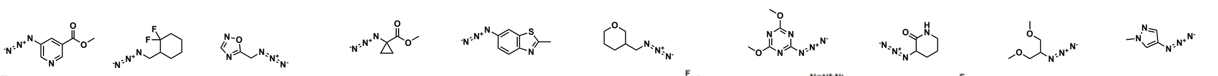

201 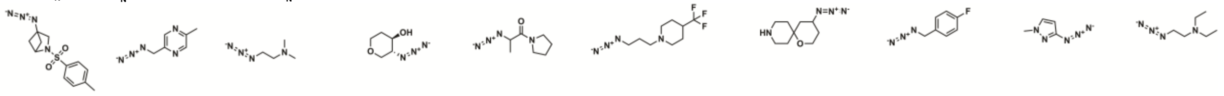

211 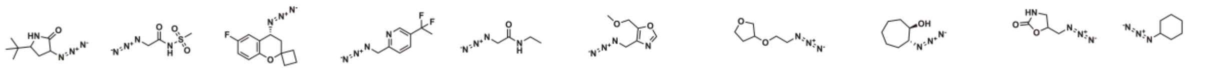

221 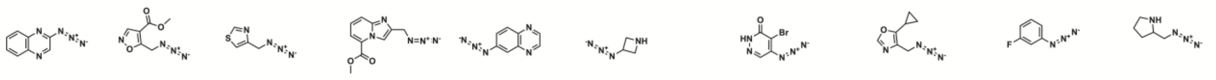

231 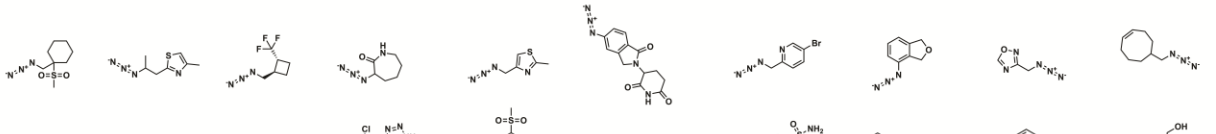

241 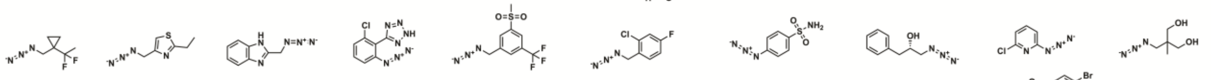

251 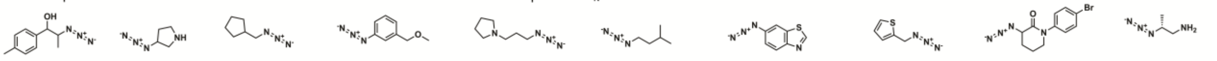

261 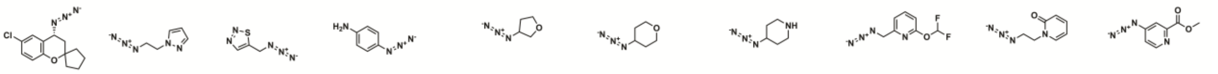

271 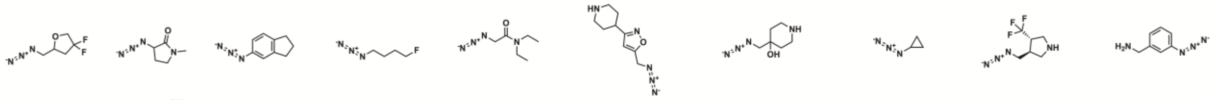

281 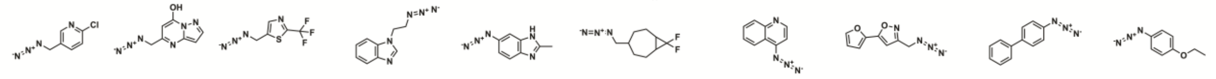

291 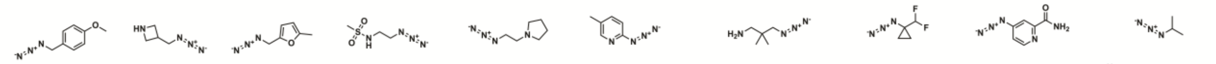

301 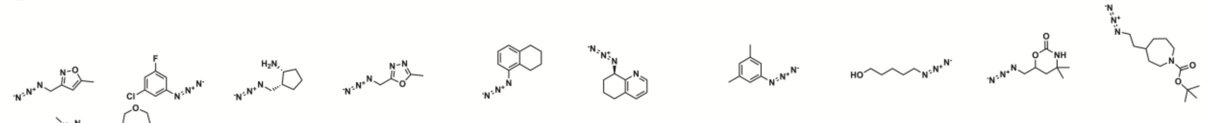

311 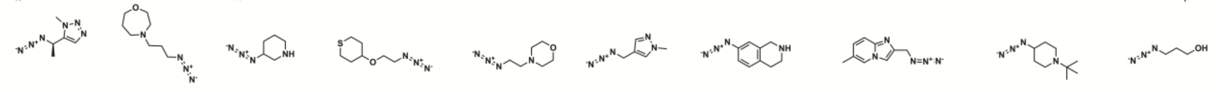

321 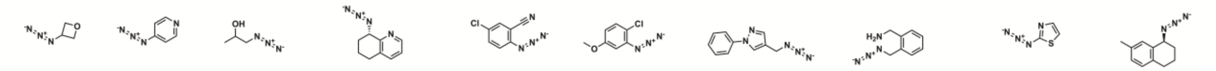

331 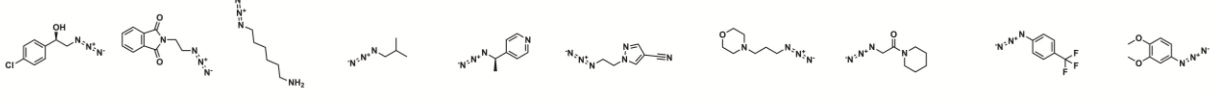

341 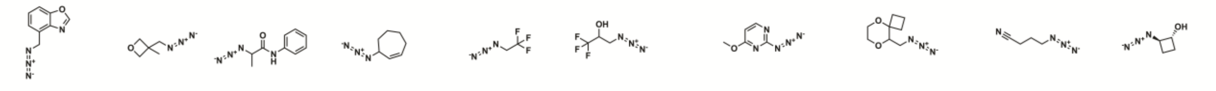

351 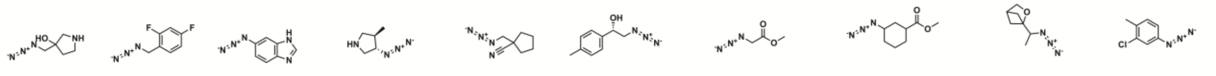

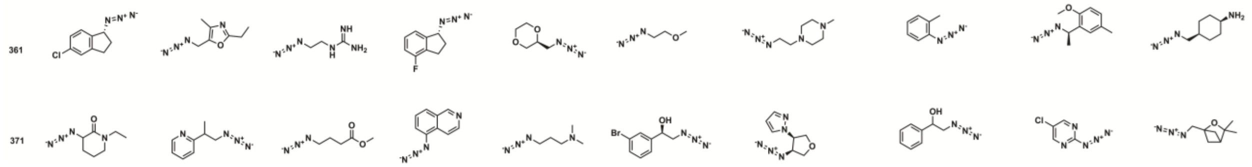

Figure S6. Structures of azide-containing small molecules from 380 compound library.

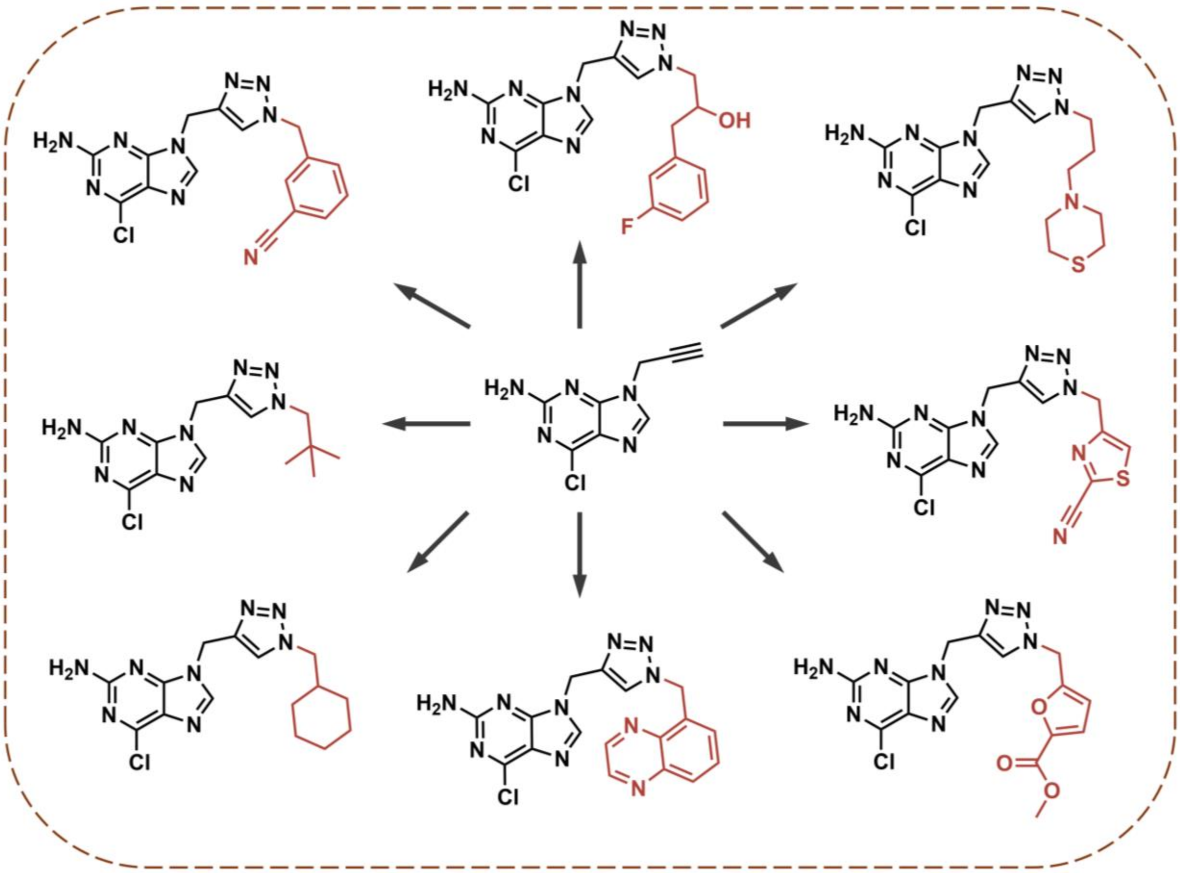

Figure S7. Chemical structures of triazole products formed from click reaction between 9-propargyl-2-amino-6-chloropurine and azide compounds 1-8 of 380 library.

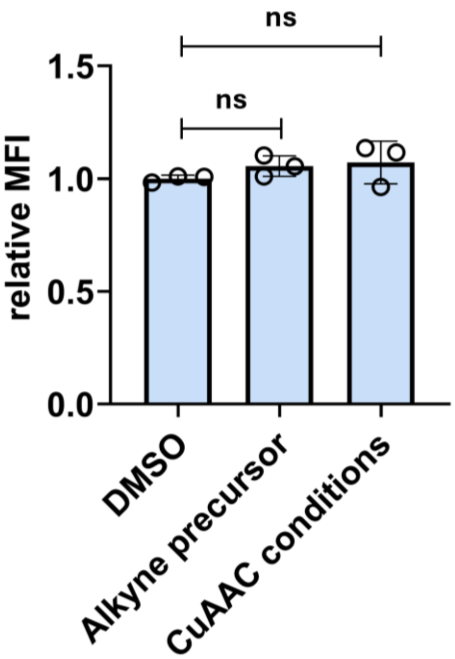

Figure S8. Flow cytometry analysis of CT26 cells treated with 1 μM 9-propargyl-2-amino-6-chloropurine or a 1:10,000 dilution of CuAAC click reagents (dilution used for Figure 4B). H-2K^d expression was measured by APC anti-mouse H-2K^d antibody. MFI is mean fluorescence intensity of the level of fluorescence relative to the DMSO control. Data are represented as mean ± SD (n=3). P-values were determined by a two-tailed *t*-test (ns = not significant).

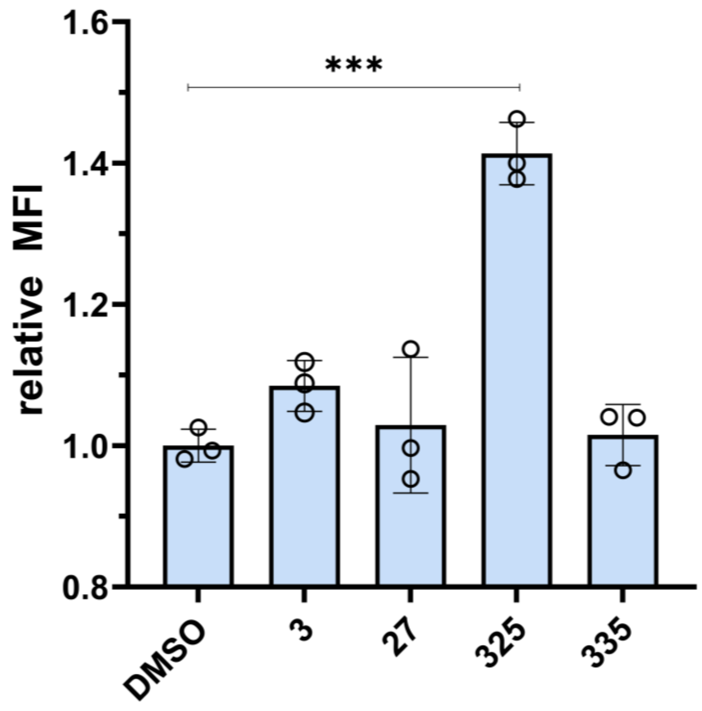

Figure S9. Flow cytometry analysis of CT26 cells treated with 1:20,000 dilution of click reaction (500 nM assuming complete conversion) mixtures containing azides 3, 27, 325, and 335. H-2K^d expression was measured by APC anti-mouse H-2K^d antibody. MFI means fluorescence intensity of the level of fluorescence relative to the DMSO control. Data are represented as mean ± SD (n=3). p-values were determined by a two-tailed *t*-test (*** $p < 0.001$).

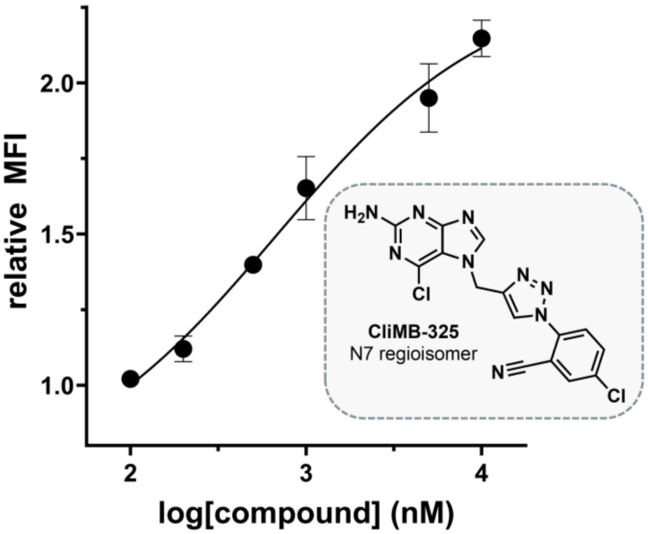

Figure S10. Dose-response curve and chemical structure of the regioisomer of **CliMB-325,** formed from a reaction between the minor N7 regioisomer of the alkyne-modified BIIB021 precursor (7-propargyl-2-amino-6-chloropurine) and 2-azido-5-chlorobenzonitrile (azide 325 from 380 compound screen). CT26 cells were treated with varying concentrations of the **CliMB-325** regioisomer. H-2K^d expression was measured by APC anti-mouse H-2K^d antibody via flow cytometry. MFI means fluorescence intensity of the level of fluorescence relative to the DMSO control. Data are represented as mean ± SD (n=3), and Boltzmann sigmoidal curves were fitted to the data using GraphPad Prism. EC_{50} values are the concentration of compound needed to achieve 50% of the maximal MHC-I surface expression levels.

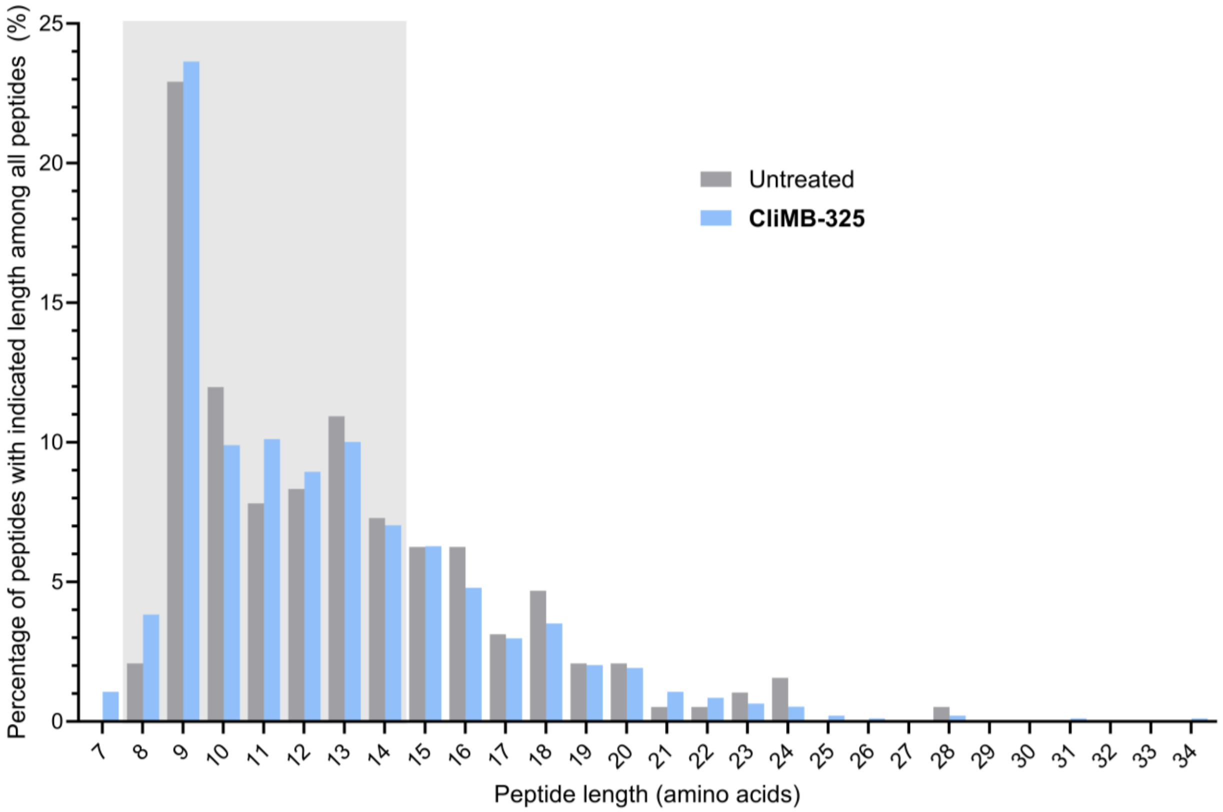

Figure S11. Length distribution of MHC-I peptides isolated by mild acid elution (MAE) from CT26 cells treated with (blue) or without (gray) **CliMB-325**. Data in displayed histograms are derived from a single cell culture, and peptides were obtained from 1 x 10^7 cells per sample. Light gray shaded area indicates the *in silico* length filter of 8 to 14 amino acids that was applied for all analyses (except for this figure). The summed fraction of 8- to 14-mers among all peptides was 64% and 66% for untreated and **CliMB-325**-treated samples, respectively.

MATERIALS

Reagents. All library compounds were purchased from either Selleck Chemicals, AK Scientific, A2B Chem, MedChemExpress, GlpBio, Cayman Chemical Company, or AmBeed. Compounds were solubilized in DMSO and stored at -20°C. Recombinant murine and human IFN-γ were purchased from PeproTech. APC-labeled antibodies against H-2K^d/H-2D^d, HLA-A,B,C, and H-2K^b bound to SIINFEKL were purchased from BioLegend. The library of 380 azide-containing small molecules were purchased from Enamine (catalog # AZD-380-X-100). For the synthesis of **CliMB-325**, 2-amino-6-chloropurine was purchased from AmBeed (catalog # A135577) and 2-azido-5-chlorobenzonitrile was purchased from Enamine (catalog # EN300-279694). Dulbecco's Modified Eagle's Medium (DMEM), Roswell Park Memorial Institute (RPMI) 1640 medium, and McCoy's 5A medium were purchased from VWR. Fetal Bovine Serum (FBS) and penicillin-streptomycin were purchased from Sigma-Aldrich.

EXPERIMENTAL METHODS

Mammalian Cell Culture. CT26 cells were cultured in RPMI 1640 media supplemented with 10% fetal bovine serum, 50 IU/mL penicillin, and 50 μg/mL streptomycin. HCT116 cells were kindly provided by Dr. Anja-Katrin Bielinsky and were cultured in McCoy's 5A media supplemented with 10% fetal bovine serum, 50 IU/mL penicillin, 50 μg/mL streptomycin, and 2 mM GlutaMAX. MC38-OVA cells were kindly provided by Dr. Mirna Perusina Lanfranca and were cultured in DMEM supplemented with 10% fetal bovine serum, 50 IU/mL penicillin, 50 μg/mL streptomycin, 50 μg/mL gentamycin, and 10 μg/mL blasticidin. B3Z cells were kindly provided by Dr. Aaron Esser-Kahn and maintained in RPMI 1640 media supplemented with 10% fetal bovine serum, 50 IU/mL penicillin, and 50 ug/mL streptomycin. All cells were cultured in T75 flasks and maintained in a humidified atmosphere of 5% CO_2 at 37°C.

Flow Cytometry-Based Assays. 1.5 x 10^4 cells were seeded in a treated 96-well plate along with indicated concentrations of library compounds at 37°C. After 48 hours, cells were washed once with PBS, removed using TrypLE™ Express Enzyme (Thermo Fisher), and transferred to a round-bottom 96-well plate. Transferred cells were centrifuged (1100 x g, 5 min) in a Thermo Scientific Jouan C4i centrifuge, and the cell pellets were resuspended and fixed in 4% formaldehyde solution for 20 minutes. The plate was centrifuged (1100 x g, 5 min) and pelleted cells were resuspended in a 1:100 dilution of indicated fluorescence antibodies in culture media for 1 hour at 4°C. Flow cytometry was performed using the following antibodies: APC anti-mouse H-2K^d/H-2D^d (clone 34-1-2S), APC anti-human HLA-A,B,C (clone W6/32), or APC anti-mouse H-2K^b bound to SIINFEKL (clone 25-D1.16). Cells were analyzed using an Attune NxT Flow Cytometer (Thermo Fisher) equipped with a 637 nm laser with 670/14 nm bandpass filter.

MTT Cell Viability Assay. 1.5 x 10^4 CT26 cells were seeded in a treated 96-well plate, either with or without compounds (BIIB021 and **CliMB-325**) at indicated concentrations at 37°C. After 48 hours, a solution of MTT in PBS (filter sterilized through a 0.2-μM filter) was added to each well to achieve a final concentration of 0.45 mg/mL. After incubating at 37°C for 2 hours, cells were centrifuged (1100 x g, 5 min) in a Thermo Scientific Jouan C4i centrifuge and the supernatant was removed. 100 μL of DMSO was added to each

well to dissolve the formation of formazan precipitate. The absorbance of the solution in each well was read at 570 nm using a BioTek Synergy H1 Microplate Reader. Wells containing no cells (only the added DMSO) were used as a negative control for viability, while untreated cells served as the positive control for 100% viability.

B3Z T Cell Activation. 1.5x10^4 MC38-OVA cells were seeded in a treated 96 well plate, either with or without compounds (BIIB021 and **CliMB-325)** at indicated concentrations at 37°C. After 48 hours, the culture media was replaced with media containing 10^5 B3Z cells, which were co-incubated with the MC38-OVA cells for 6 hours. Cells were centrifuged (1100 x g, 5 min) in a Thermo Scientific Jouan C4i centrifuge and the supernatant was removed. Lysis buffer containing 0.2% saponin, 500 mM CPRG reagent, 20 mM $MgCl_2$, and 100 mM β-mercaptoethanol in 1X PBS was added to each well. After 45 minutes, absorbance at 570 nm was recorded using a BioTek Synergy H1 Microplate Reader.

Molecular Docking Studies. Conformational predictions of **CliMB-325** in Hsp90 were performed using RosettaLigand using the crystal structure of BIIB021 bound to Hsp90 (PDB ID: 3qdd).[1-4] Native crystal structure was prepared for docking by removing all water molecules and co-crystallized ligands. PyMOL was used for visualization of the docking results.

Mild Acid Elution (MAE) of MHC-I-Bound Peptides. The MAE protocol was adapted from a previously published protocol.[5] MAEs from CT26 cells were performed with 1 x 10^7 CT26 cells per sample. Cells were washed three times with PBS then were treated for 90 seconds with MAE buffer. The MAE buffer consisted of 131 mM citric acid, 66 mM Na_2HPO_4, and 150 mM NaCl adjusted to pH 3.3 with NaOH. Following treatment with the MAE buffer, the eluted peptide solution was centrifuged (4000 x g, 5 min) in a Thermo Scientific Jouan C4i centrifuge. The peptide-containing supernatant was collected and acidified with 0.1% TFA. The obtained peptide solution was further purified on Oasis HLB columns (barrel size 1 cm^3, 30 mg of sorbent; Waters, product no.: WAT094225) prerinsed with 100% acetonitrile (MeCN)/0.1% TFA. After equilibration with 100% H_2O/0.1% TFA, sample loading, and washing with 95% MeCN/0.1% TFA, peptides were eluted with 60% MeCN/0.1% TFA. The eluate was filtered through a 15 mL Amicon ultrafilter device with 3 kDa molecular weight cutoff (Merck Millipore, Cat.-No. UFC901024) then lyophilized to dryness using a Labconco Freezone 4.5 L (- 84°C) lyophilizer.

Liquid Chromatography. Peptide separation was carried out on a Vanquish Neo UHPLC system using a trap-and-elute setup. An Aurora Frontier™ TS C18 column (IonOpticks; 60 cm × 75 μm, 1.7 μm particles) was used for peptide separation. The mobile phases used were: Phase A — 0.1% formic acid (FA) in water; Phase B — 80% acetonitrile (ACN) with 0.1% FA in water. A 40-minute gradient was applied at 0.3 μl/min: 15% to 50% MPB from 0.1 to 40 min, followed by 50% to 99% mobile phase buffer B (MPB) from 40.1 to 42 min, held at 99% MPB until 50 min, and re-equilibrated at 1% MPB for 35 minutes.

Mass Spectrometry Data Acquisition. Data were acquired using an Orbitrap Astral mass spectrometer in a DDA mode. For MS1 scans, the Orbitrap resolution was set at 120,000 with an AGC target of 100%. MS1 spectra were recorded over an m/z range of 350–1350, with a maximum injection time of 50 ms. The isolation window for MS2

precursor selection was set to 1.2 m/z. Up to 30 scans were acquired per MS1 cycle for precursor ions with intensities greater than 5.0×10^3 and charge states ranging from 2 to 6. MS2 fragmentation was performed with HCD at 25% collision energy and a maximum injection time of 25 ms. Dynamic exclusion was turned on with a duration of 20 seconds.

DDA Data Analysis. Spectra were converted to mzXML using a modified version of ReAdW.exe. Mass spectra were processed using a COMET-based software pipeline and searched against the mouse UniProt database (downloaded on October 3rd, 2024). Database searches were performed using a precursor ion tolerance of 50-ppm and 0.02 Da fragment ion tolerance. No enzyme specificity was specified for peptide identification. Carbamidomethylation of cysteine residues (+57.021 Da) were set as static modifications, while oxidation of methionine residues (+15.995 Da) was set as a variable modification. Peptide-spectrum matches (PSMs) were adjusted to a 1% false discovery rate (FDR) using standard target-decoy approaches.[6] Sequenced peptides were further filtered based on length (8–14 amino acids) to ensure likelihood of MHC-I binding. The obtained peptide list was input into NetMHCpan 4.1 database to predict binding compatibility with the MHC-I allotypes expressed by CT26 cells (H-2-Dd, H-2-Kd, and H-2-Ld).[7] Prediction score was reported by NetMHCpan 4.1, and downstream peptide analysis was only performed on peptides which were modeled to bind with a prediction score of 2.0 or lower and thus expected to be putative binders of MHC-I molecules.

SYNTHESIS AND CHARACTERIZATION

Scheme S1. Synthesis of 9-propargyl-2-amino-6-chloropurine

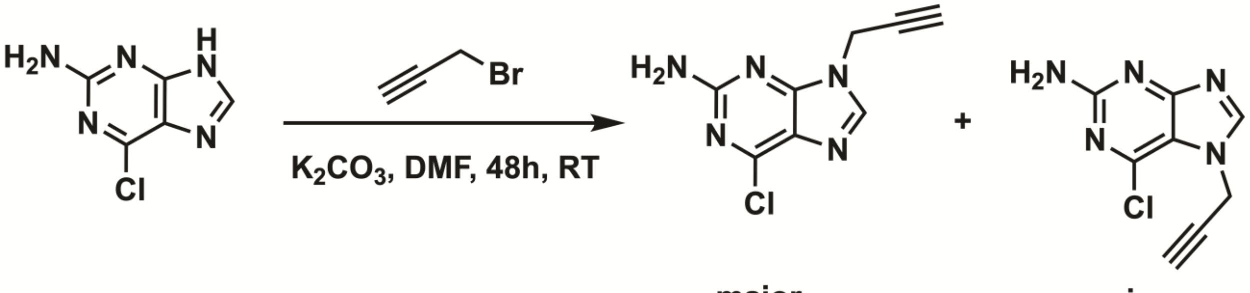

major minor

9-propargyl-2-amino-6-chloropurine was synthesized based on literature procedure.[8] 2-amino-6-chloropurine (3.0 g, 1 eq) was suspended DMF (50 mL) followed by addition of anhydrous K_2CO_3 (2.934 g, 1.2 eq) and stirring under N_2 atmosphere for 1 hour. After this time, propargyl bromide (1.894 g, 0.9 eq) was added and stirred for 48 hours under N_2 atmosphere at room temperature. DMF was evaporated at 60°C under high vacuum to afford a yellowish-white powder. A 1:2 ratio of minor and major compound was produced as determined by NMR. The crude material was purified by reverse-phase preparative high-performance liquid chromatography (RP-HPLC) equipped with Waters 1525 with a 2489 UV/Visible Detector monitoring at 311 nm wavelength, on a Phenomenex Luna Omega 5 μM Polar C18 250 x 21.2 mm column using gradient elution with using H_2O/MeOH with 0.1% TFA at 10 mL/min. The HPLC fractions of the major compound were concentrated under reduced pressure using a rotary evaporator, then lyophilized to dryness using a Labconco Freezone 4.5 L (- 84°C) lyophilizer and characterized by NMR which matched with the reported compound.[9] This product was analyzed for purity using RP analytical HPLC equipped with Waters 1525 with a 2489 UV/Visible Detector monitoring at 311 nm wavelength, on a Phenomenex Luna 5 μM C18(2) 250 x mm column using gradient elution with using H_2O/MeOH with 0.1% TFA at 1 mL/min. The major product was used for click chemistry.

Analytical HPLC chromatogram of 9-propargyl-2-amino-6-chloropurine

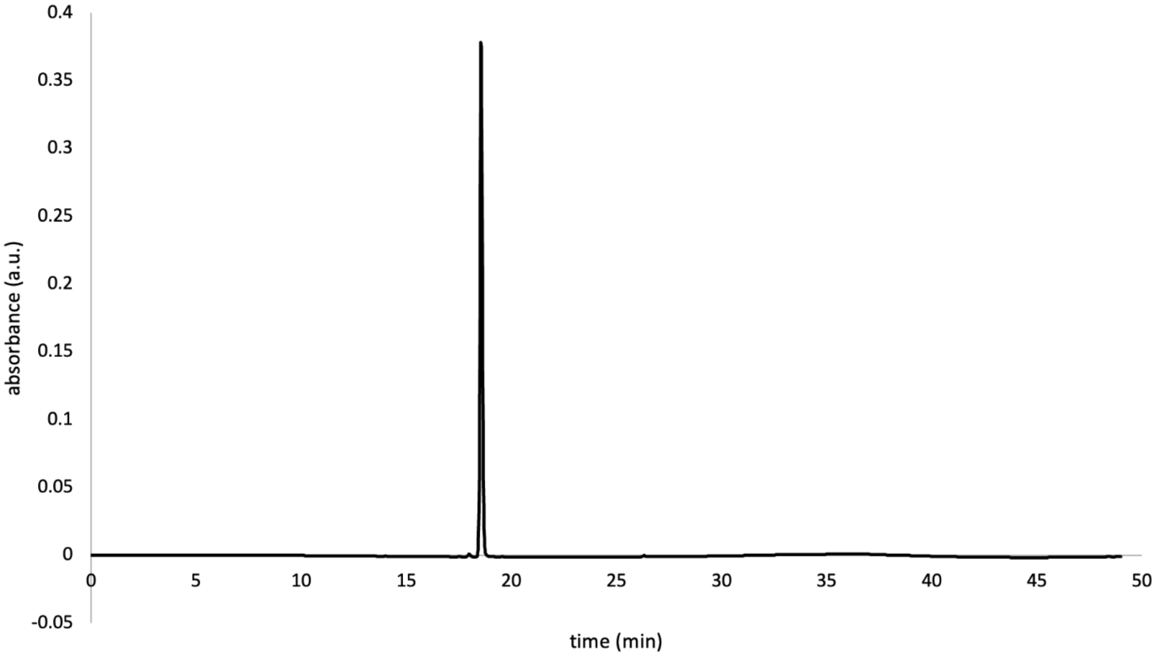

ESI High Resolution Mass Spectrum for 9-propargyl-2-amino-6-chloropurine

m/z calculated for $C_8H_6ClN_5$ $[M+H]^+$ 208.0385, found 208.0388.

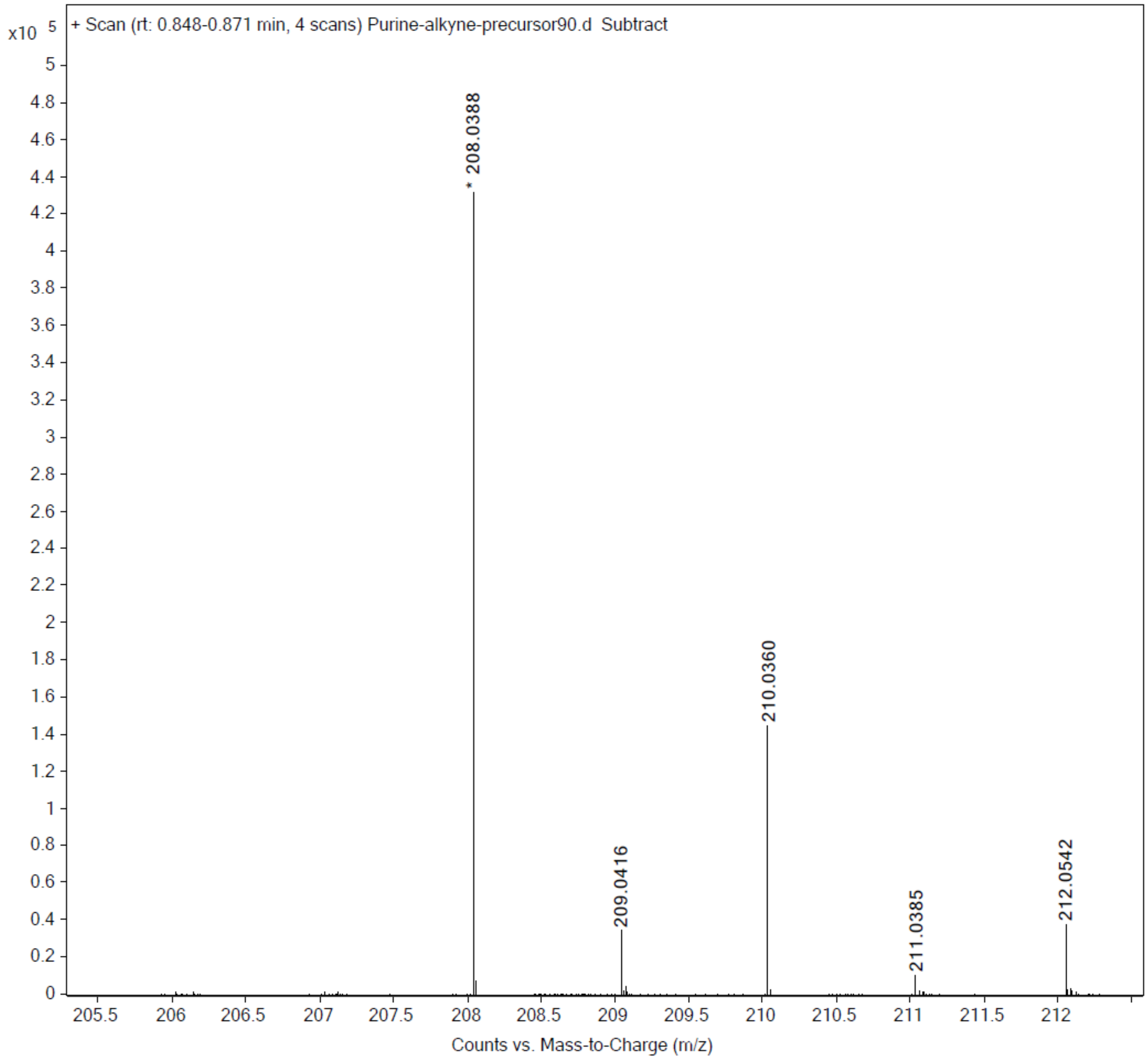

NMR of 9-propargyl-2-amino-6-chloropurine

6-chloro-9-(prop-2-yn-1-yl)-9H-purin-2-amine. White solid;[9] ^1H NMR (600 MHz, DMSO-*d*$_6$) δ 8.18 (s, 1H, 8-H), 7.02 (brs, 2H, $-NH_2$), 4.93 (d, 2H, J=1 Hz, $-CH_2$), 3.48 (t, 1H, J = 1 Hz, C≡CH).

Scheme S2. High-Throughput Synthesis of Triazole-Containing BIIB021 Derivatives

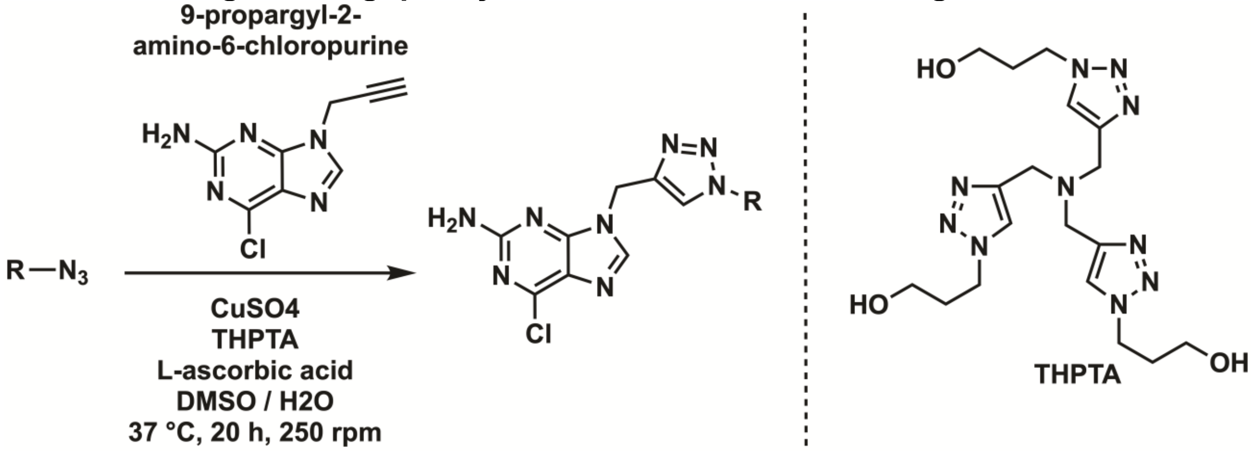

Triazole analogs were synthesized based on literature procedure.[10] Azide solutions from the azide library in Plates 1-5 were initially at a concentration of 100 mM in DMSO. Azides were added in each well of a 96-well plate at a concentration of 10 mM. To each well of this newly loaded plate, L-ascorbic acid solution was added to a concentration of 40 mM along with 10 mM of 9-propargyl-2-amino-6-chloropurine (synthesis shown in *Scheme S1*) and 2 mM of $CuSO_4$/THPTA in a solution of DMSO and water at a 3:2 ratio to a total volume of 100 µL. The plates were sealed and swirled at 250 rpm and 37°C for 20 hours to afford the corresponding triazole product in each well.

Scheme S3. Synthesis of CliMB-325.

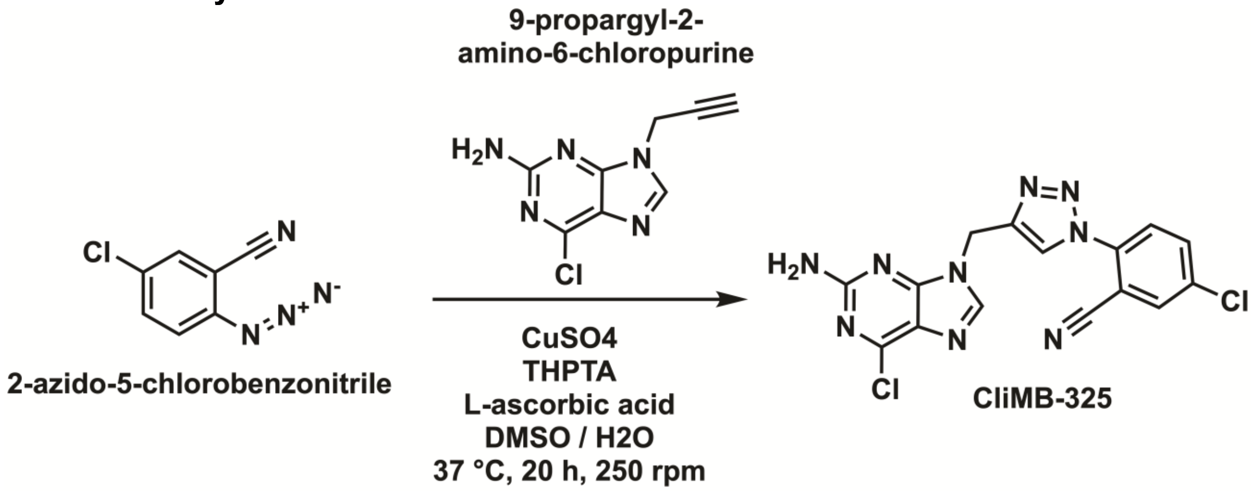

In a 50 mL conical tube, the following reagents were added: 10 mM of 2-azido-5-chlorobenzonitrile (azide #325 from 380 compound screen), 40 mM of aqueous L-ascorbic acid, 10 mM of 9-propargyl-2-amino-6-chloropurine (synthesis shown in *Scheme S1*), 2 mM of aqueous $CuSO_4$/THPTA solution, in a 3:2 ratio of DMSO to water at a total volume of 15 mL. The tube was swirled at 250 rpm and 37°C for 20 hours to yield **CliMB-325**. The compound was purified by reverse-phase preparative high-performance liquid chromatography (RP-HPLC) equipped with Waters 1525 with a 2489 UV/Visible Detector monitoring at 311 nm wavelength, on a Phenomenex Luna Omega 5 µM Polar C18 250 x 21.2 mm column using gradient elution with using H_2O/MeCN with 0.1% TFA at 10 mL/min. The HPLC fractions of the desired purified product were concentrated under reduced pressure using a rotary evaporator, then lyophilized to dryness using a Labconco Freezone 4.5 L (- 84°C) lyophilizer. The product was analyzed for purity using RP analytical HPLC equipped with Waters 1525 with a 2489 UV/Visible Detector monitoring at 311 nm wavelength, on a Phenomenex Luna 5 µM C18(2) 250 x mm column using gradient elution with using H_2O/MeCN with 0.1% TFA at 1 mL/min. The final product was stored at -20°C until further use, and stocks were made at 10 mM in DMSO.

Analytical HPLC Chromatogram of **CliMB-325**

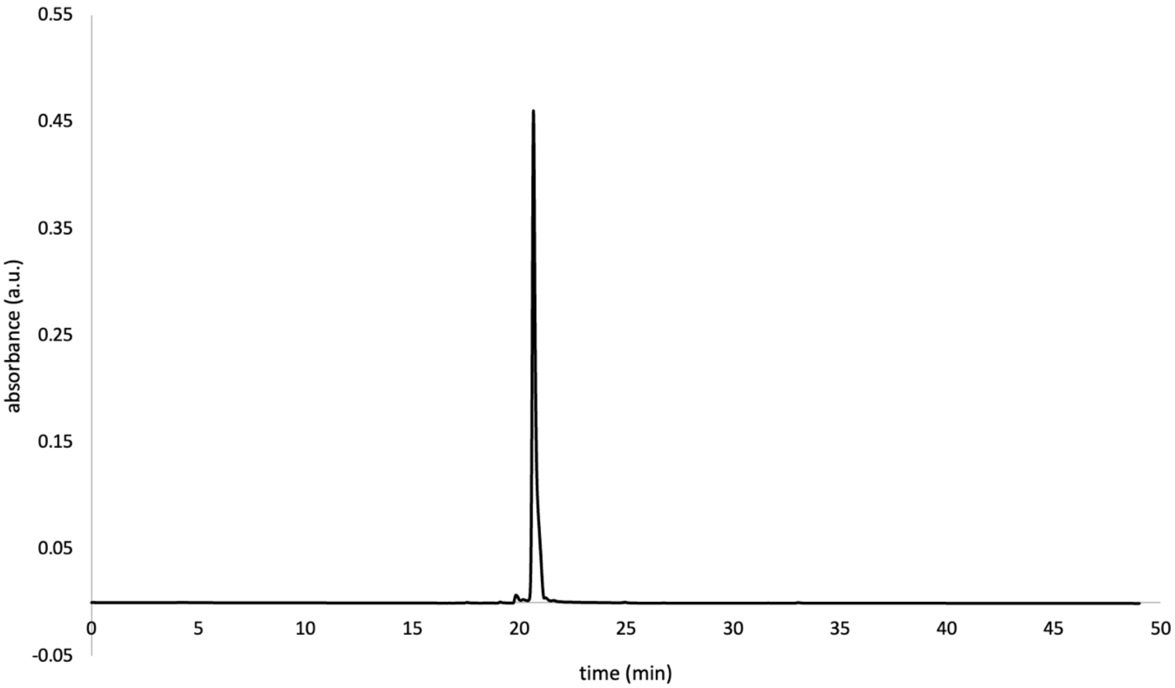

ESI High Resolution Mass Spectrum for **CliMB-325**

m/z calculated for $C_{15}H_{10}Cl_2N_9$ $[M+H]^+$ 386.0431, found 386.0439.

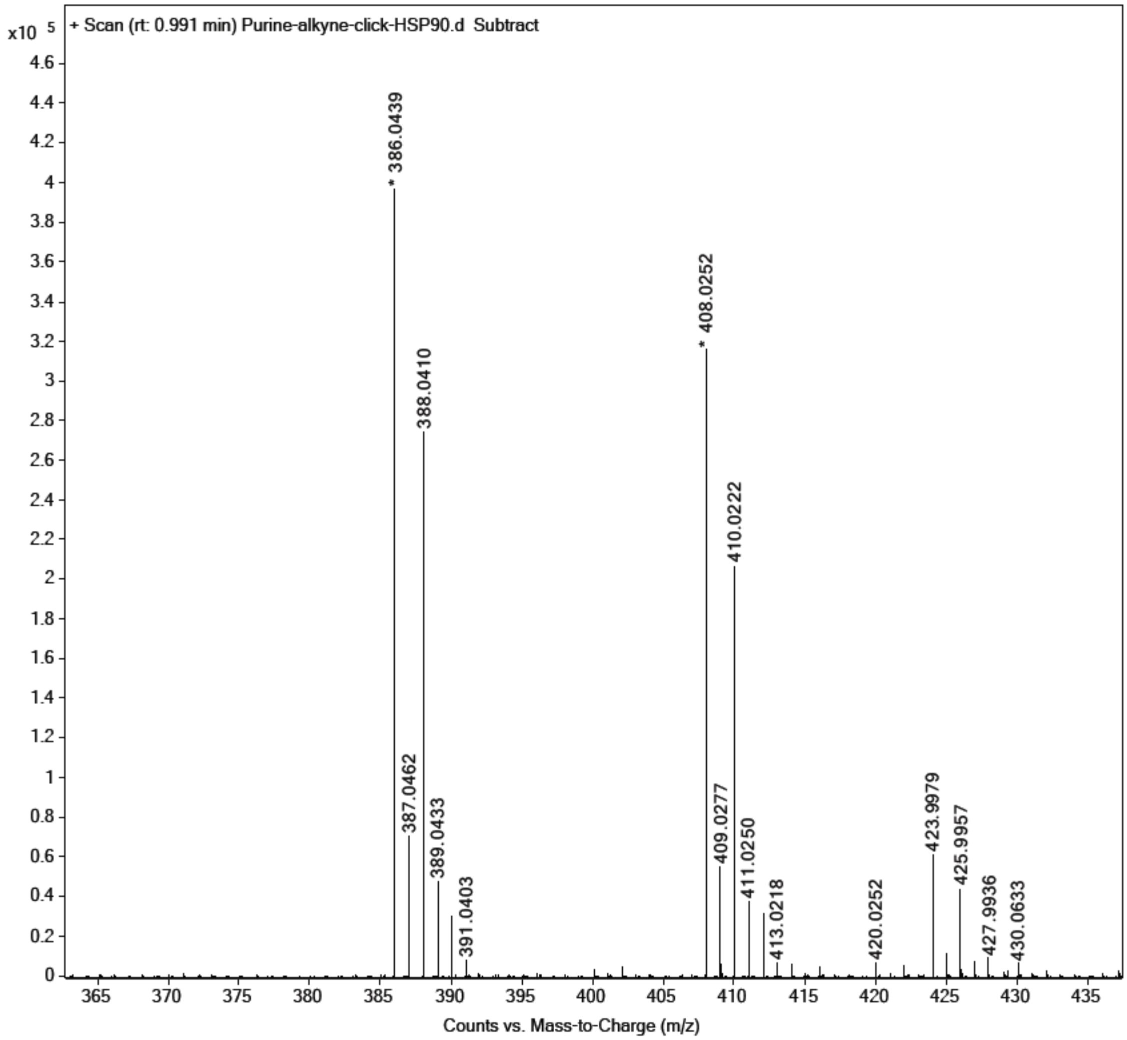

^{1}H NMR of **CliMB-325**

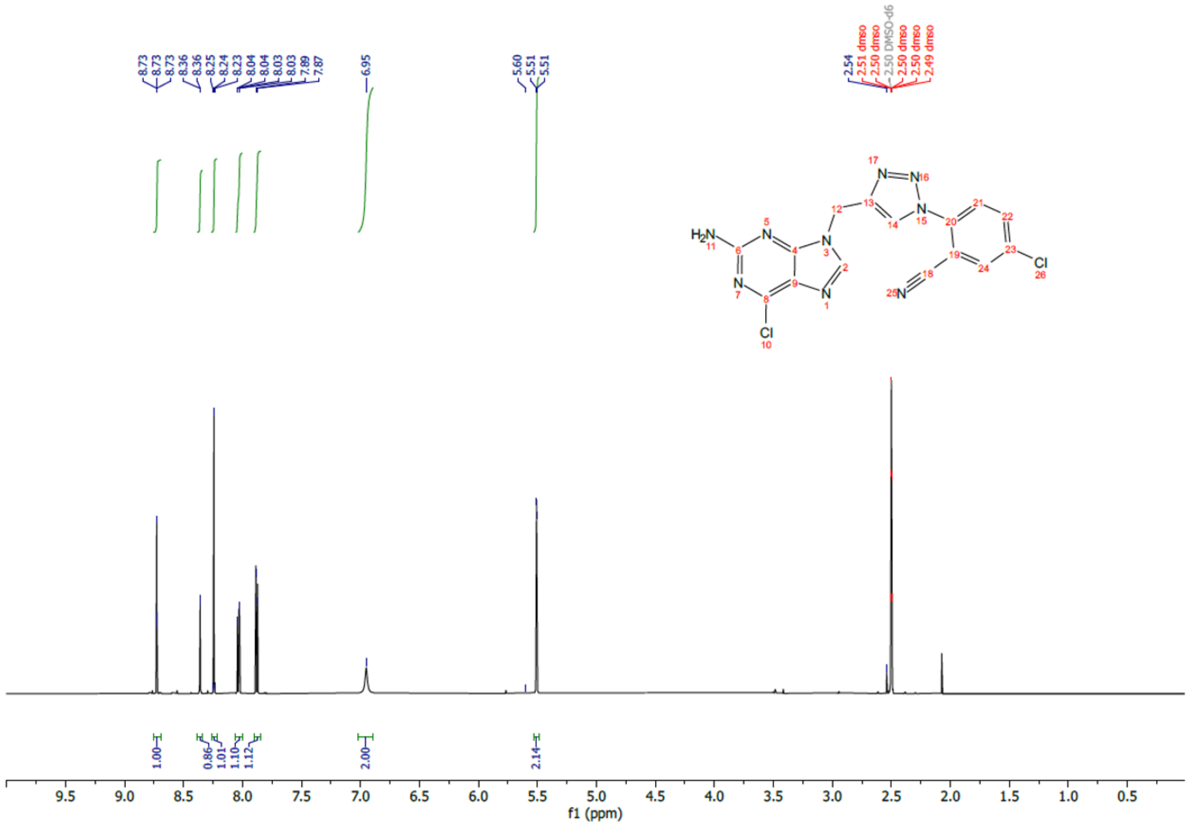

^{13}C NMR of **CliMB-325**

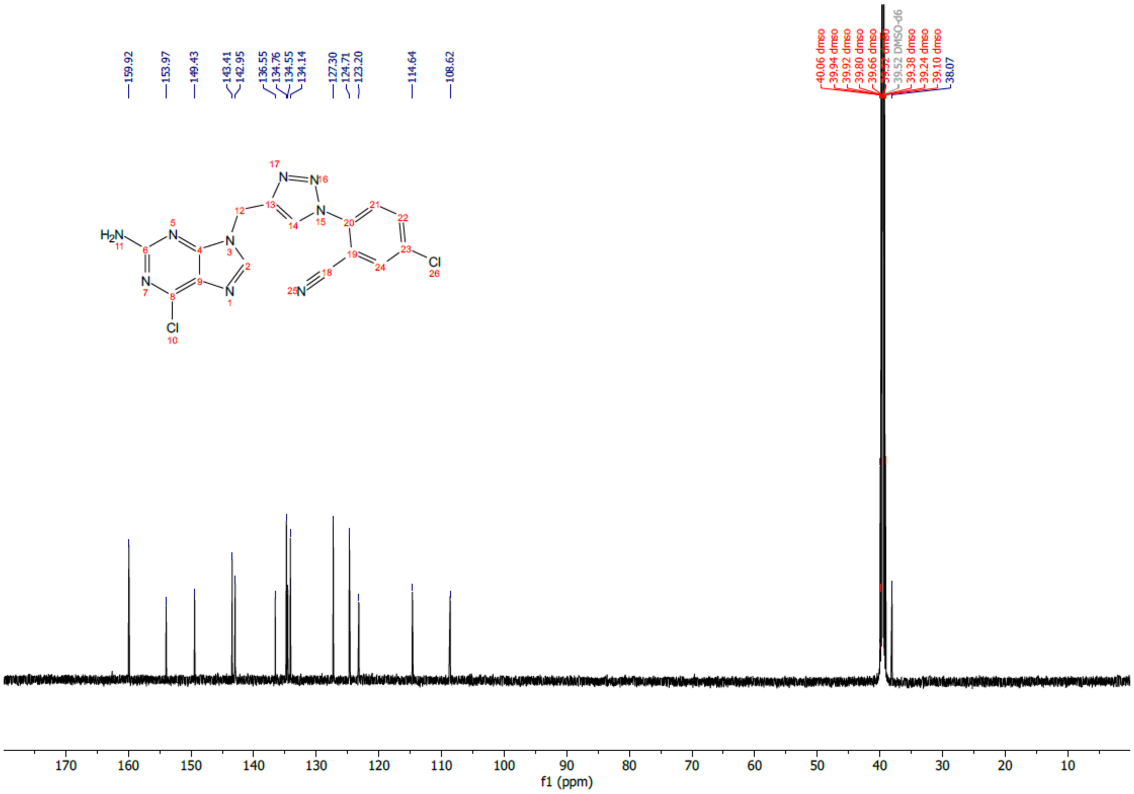

2-(4-((2-amino-6-chloro-9H-purin-9-yl)methyl)-1H-1,2,3-triazol-1-yl)-5-chlorobenzonitrile. White solid; ^{1}H NMR (600 MHz, DMSO-d_6) δ 8.73 (s, 1H, 8-H), 8.36 (d, 1H, J= 1 Hz, Ar-H), 8.24 (s, 1H, triazole-H), 8.04 (dd, 1H, J= 3.6 Hz, Ar-H), 7.88 (d, 1H, J= 3.6 Hz, Ar-H), 6.95 (brs, 2H, -NH), 5.51 (s, 2H, -CH_2); ^{13}C NMR (150 MHz, DMSO-d_6) δ 159.9, 153.9, 149.4, 143.4, 142.9, 136.5, 134.7, 134.5, 134.1, 127.3, 124.7, 123.2, 114. 6, 108.6, 38.0

REFERENCES

(1) Combs, S. A.; Deluca, S. L.; Deluca, S. H.; Lemmon, G. H.; Nannemann, D. P.; Nguyen, E. D.; Willis, J. R.; Sheehan, J. H.; Meiler, J. Small-molecule ligand docking into comparative models with Rosetta. *Nat Protoc* **2013**, 8 (7), 1277-1298. DOI: 10.1038/nprot.2013.074

(2) DeLuca, S.; Khar, K.; Meiler, J. Fully Flexible Docking of Medium Sized Ligand Libraries with RosettaLigand. *PLoS One* **2015**, 10 (7), e0132508. DOI: 10.1371/journal.pone.0132508

(3) Kothiwale, S.; Mendenhall, J. L.; Meiler, J. BCL::Conf: small molecule conformational sampling using a knowledge based rotamer library. *J Cheminform* **2015**, 7, 47. DOI: 10.1186/s13321-015-0095-1

(4) Lyskov, S.; Chou, F. C.; Conchuir, S. O.; Der, B. S.; Drew, K.; Kuroda, D.; Xu, J.; Weitzner, B. D.; Renfrew, P. D.; Sripakdeevong, P.; et al. Serverification of molecular modeling applications: the Rosetta Online Server that Includes Everyone (ROSIE). *PLoS One* **2013**, 8 (5), e63906. DOI: 10.1371/journal.pone.0063906

(5) Sturm, T.; Sautter, B.; Worner, T. P.; Stevanovic, S.; Rammensee, H. G.; Planz, O.; Heck, A. J. R.; Aebersold, R. Mild Acid Elution and MHC Immunoaffinity Chromatography Reveal Similar Albeit Not Identical Profiles of the HLA Class I Immunopeptidome. *J Proteome Res* **2021**, 20 (1), 289-304. DOI: 10.1021/acs.jproteome.0c00386

(6) Elias, J. E.; Gygi, S. P. Target-decoy search strategy for increased confidence in large-scale protein identifications by mass spectrometry. *Nat Methods* **2007**, 4 (3), 207-214. DOI: 10.1038/nmeth1019

(7) Reynisson, B.; Alvarez, B.; Paul, S.; Peters, B.; Nielsen, M. NetMHCpan-4.1 and NetMHCIIpan-4.0: improved predictions of MHC antigen presentation by concurrent motif deconvolution and integration of MS MHC eluted ligand data. *Nucleic Acids Res* **2020**, 48 (W1), W449-W454. DOI: 10.1093/nar/gkaa379

(8) Nagapradeep, N.; Verma, S. Characterization of an unprecedented organomercury adduct via Hg(II)-mediated cyclization of N9-propargylguanine. *Chem Commun (Camb)* **2011**, 47 (6), 1755-1757. DOI: 10.1039/c0cc03123b

(9) Lindsell, W. E.; Murray, C.; Preston, P. N.; Woodman, T. A. J. Synthesis of 1,3-diynes in the purine, pyrimidine, 1,3,5-triazine and acridine series. *Tetrahedron* **2000**, 56 (9), 1233-1245.

(10) Xin, Y.; Liu, S.; Liu, Y.; Qian, Z.; Liu, H.; Zhang, B.; Guo, T.; Thompson, G. J.; Stevens, R. C.; Sharpless, K. B.; et al. Affinity selection of double-click triazole libraries for rapid discovery of allosteric modulators for GLP-1 receptor. *Proc Natl Acad Sci U S A* **2023**, 120 (11), e2220767120. DOI: 10.1073/pnas.2220767120
